# Costs and cost effectiveness of the use of telerehabilitation training for upper limb function in people after stroke: A systematic review

**DOI:** 10.1177/02692155261441561

**Published:** 2026-05-02

**Authors:** Daim Syukriyah, Jemma Perks, Phil McBride, Philip Clatworthy, Helen Dawes, Maedeh Mansoubi, Tristan Snowsill, Gordon Taylor, Claire Hulme

**Affiliations:** 1 Department of Health and Community Sciences, 151673 University of Exeter, Exeter, UK; 2 Department of Public Health and Sport Sciences, 3286 University of Exeter, Exeter, UK; 3 1982 North Bristol NHS Trust, Bristol, UK; 4 Medical School, Faculty of Health and Life Sciences, University of Exeter, UK; 5 NIHR Exeter Biomedical Research Centre (BRC) and Health Research Collaboration (HRC), University of Exeter, UK

**Keywords:** Stroke, upper extremity, telerehabilitation, economic evaluation, costs, cost-effectiveness analysis

## Abstract

**Objective:**

To review the costs and cost-effectiveness of telerehabilitation training for upper limb function in people after stroke.

**Data Sources:**

MEDLINE, EconLit, and EMBASE databases were searched for studies published between 2013 and 16 March 2026.

**Methods:**

The review included studies of technology-based rehabilitation programmes for individuals after stroke that evaluated costs or cost-effectiveness. Titles and abstracts were screened, and data were independently extracted by two researchers. Study quality was appraised using the Critical Appraisal Skills Programme (CASP) checklist. Findings were synthesised narratively.

**Results:**

Fifteen studies including 963 participants were included. Two studies conducted cost-effectiveness analyses, eight reported cost analyses, and six reported the use of a preference-based measure recommended for cost-effectiveness studies (all used the EQ-5D), with some studies contributing to more than one category. Evidence on costs and cost-effectiveness was mixed, but several studies suggested potential cost savings. Reporting of EQ-5D outcomes was inconsistent across studies.

**Conclusions:**

Evidence on the costs of telerehabilitation training for upper limb function primarily focused on therapist time and equipment costs. Few studies included costs associated with equipment maintenance, depreciation, or the need for internet-enabled devices. Reductions in therapist time could offset or exceed technology costs. Further research is needed to evaluate the longer-term costs and cost-effectiveness of telerehabilitation in this population.

## Background

Stroke recovery is facilitated by rehabilitation and can continue for years, although the most rapid recovery occurs in the first weeks and months. There are over one million stroke survivors in the UK, with around 100,000 new stroke cases each year; estimates have placed the annual cost attributed to stroke in the UK at £26 billion.^1^ Responding to this demand, the NHS in the UK has aimed to provide more rehabilitation at home early after stroke, potentially using telerehabilitation, which refers to the remote delivery of rehabilitation services via information and communication technologies, such as video conferencing, apps, sensors, or platforms connecting patients with clinicians.^2^ Virtual reality (VR) and other technology-based interventions may form part of telerehabilitation when they are embedded within structured rehabilitation programmes and include therapeutic input.

This review builds on evidence from a Cochrane Review, which evaluated whether the use of telerehabilitation improves the ability to perform activities of daily living among stroke survivors.^3^ Comparisons of telerehabilitation and in-person therapy suggested that telerehabilitation was not inferior, and some included studies reported that telerehabilitation was less expensive. However, there was a lack of detailed information about cost-effectiveness.^3^

This review focuses on upper limb function among early stroke survivors with functional arm and hand impairments. Problems with upper limb function are very common after a stroke,^4^ and the use of virtual reality (VR) and interactive video gaming may be beneficial in improving upper limb function and activities of daily living when used as an adjunct to usual care (to increase overall therapy time).^3^

This study aims to review evidence of technology-based training for upper limb function in people after stroke, including interventions such as virtual reality applications, robotic-aided devices, video games, telephone-based therapy, and other computer technologies. The review addresses three questions:

- What are the costs associated with implementing and delivering telerehabilitation training for upper limb function in people after stroke?
- To what extent is telerehabilitation training for upper limb function in people after stroke cost-effective?
- What is the preference-based utility values (numerical scores reflecting health-related quality of life) for people after stroke undergoing telerehabilitation?

The latter question is important for cost-effectiveness analysis, which compares the costs and health outcomes of alternative interventions, services, or programmes. In the UK, the National Institute for Health and Care Excellence recommends that cost-effectiveness analyses be undertaken using quality-adjusted life years (QALYs), a single metric that combines length and quality of life into one measure of health gain. One QALY represents one year of life in perfect health. QALYs are derived from preference-based measures, such as the EQ-5D instruments, which assign utility values to different health states.^5^

## Methods

This systematic review used the Preferred Reporting Items for Systematic Reviews and Meta-Analyses (PRISMA) guidelines to incorporate all relevant studies. The study protocol was published on the PROSPERO International Prospective Register of Systematic Reviews with a reference number of CRD42023447176 on August 1, 2023.

A systematic search was undertaken using MEDLINE, EconLit, and EMBASE to identify studies published from 2013 to 16 March 2026. Two independent researchers carried out the search between 13 and 16 March 2026. Search terms were developed to reflect the key concepts of stroke, upper limb rehabilitation, and technology-based or telerehabilitation interventions. Synonyms within each concept were combined using the Boolean operator ‘OR’, and the concepts were combined using ‘AND’. The full list of search terms is presented in Table 1, and the full MEDLINE search strategy is provided in Supplementary Appendix 1.

**Table 1.** Keywords used in the identification search strategy.

Concept	Keywords in search strategy
Stroke	stroke; cerebrovascular accident*; cerebrovascular insult*; cerebrovascular infarct*
Rehabilitation	rehabilitation; neurorehabilitation; physiotherapy.
Upper limb	upper limb; upper extremity; hand; forearm.
Technology-based / telerehabilitation	telerehabilitation; telemedicine; videoconferencing; virtual reality; augmented reality; video games; exergames; commercial gaming platforms (e.g., Kinect, Nintendo Wii); communication platforms (e.g., Zoom, Skype, Microsoft Teams); computer-assisted instruction.

Studies were included if (1) they were written in English and published since 2013; (2) the papers reported any form of interventional economic evaluation (including economic modelling), cost study, or reported the use of a preference-based measure; (3) participants were explicitly described as people after stroke or stroke survivors and had functional arm and hand impairments, as defined by the original study authors; (4) the intervention was telerehabilitation training; and (5) there was a comparator group that received usual care or another rehabilitation training. Literature reviews, concept papers, opinion pieces, or editorials were excluded from the review.

EndNote was used for reference management and removal of duplicates. Two independent reviewers read and examined all titles and abstracts of the extracted articles and reviewed them against the eligibility criteria. Disagreements were resolved through discussion, with one study was discussed with a third reviewer where necessary.

During full-text retrieval, short summaries, abstracts, study protocols, and conference posters were excluded. Eligible studies proceeded to data extraction using a bespoke data extraction table. One independent researcher extracted information including the study design, country of study, sample size, and participant characteristics (including age and time since stroke onset), duration of the intervention, details of the intervention and comparator groups, costs, cost-effectiveness outcomes, preference-based measures, study strengths and limitations, and conclusions. The second researcher reviewed the data extraction table alongside the included papers and provided input to refine the table.

Independent critical appraisal was undertaken using the appropriate Critical Appraisal Skills Programme (CASP) checklist, selected according to study design. The CASP questions focus on study validity, how costs and outcomes were assessed and compared where applicable, and whether the results may be transferable to other settings.^6^ Two reviewers independently assessed included studies and disagreements were resolved through discussion. A structured summary of the appraisal is presented in Supplementary Appendix 2.

## Results

A total of 15 studies met the criteria and were included in the review (Figure 1). Studies spanned a range of geographical locations, with two studies from the USA (13.33%), two from Spain (13.33%), two from China (13.33%), and two from Italy (13.33%). The remaining studies were from the UK, Germany, Australia, Singapore, Mexico, and Taiwan; one study did not focus on a single country setting but instead included three countries: Denmark, Norway, and Belgium.^7^ Details are shown in Table 2. In total, across the 15 studies, 963^a^ participants were included, all of whom had experienced a stroke; 67% had ischaemic or haemorrhagic stroke, and 55% were male. The average age of participants was 63.15 years, ranging from 18 to 90 years. Time since stroke onset varied from seven days to 13.4 years, with 33.33% within 6 months post-stroke.^8,9^ Eleven studies reported randomised controlled trials (73.33%). Two studies used “before and after” study designs (13.33%),^10,11^ while the remaining two used retrospective studies with case matching based on patients’ characteristics.^8,12^ Four papers reported interventions for both upper and lower limb^8,12,13,14^; although these are included for completeness, none reported costs or cost-effectiveness separately for upper and lower limb outcomes.

**Figure 1.**
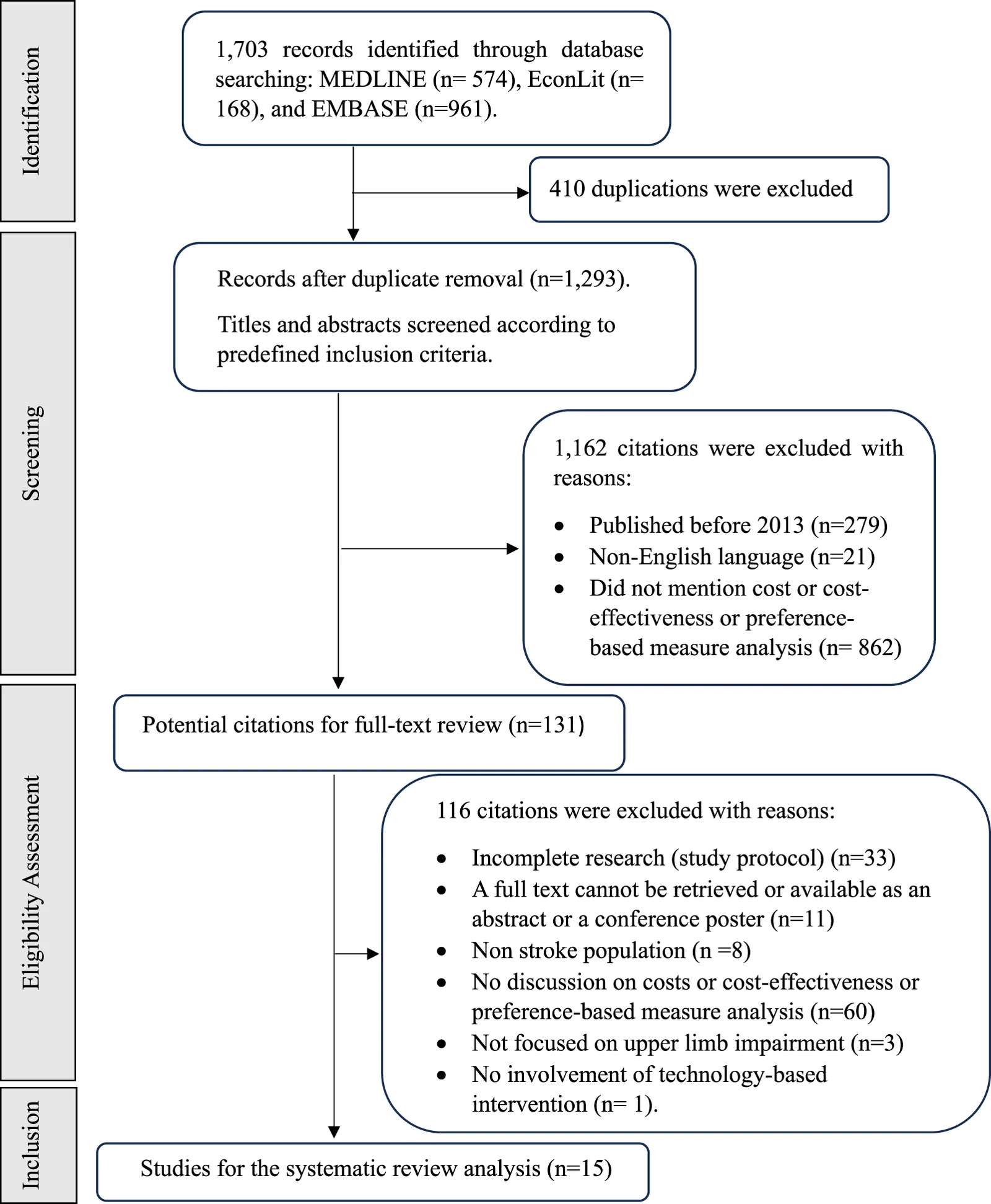
Study selection process: PRISMA diagram for the literature search and study selection.

**Table 2.** Characteristics of the telerehabilitation interventions.

Author, year, country	Aim	Study design & population	Treatment/control	Methods (costs, cost effectiveness, preference-based measures)	Results	Conclusion	Limitations
Adie et al. 2017. UK	To investigate the efficacy of using the Nintendo Wii Sports™ (Wii™) to improve affected arm function after stroke.	Cost effectiveness analysis alongside an RCT N = 209 Mean age 67.3 ±13.4 Stroke in previous 6 months	A 45-min daily exercise at home in a seated position using (i) Wii or (ii) arm exercises over 6 weeks	Cost effectiveness analysis using EQ-5D-3L. ICERs reported	EQ-5D-3L values not reported (only VAS). No difference in QALYs, higher costs in Wii group (£1106 (SD £1656) vs £730 (SD £829). The probability of Wii being dominated is 0.866.	Wii was not cost effective compared to arm exercises in home-based rehabilitation for stroke survivors with arm weakness.	No duration of exercising time was collected which might affect the results.
Aguirre-Ollinger et al. 2024. Singapore	To assess the feasibility, safety, and efficacy of home-based robot-assisted therapy (RAT) using a 2D-planar end-effector robotic arm, the H-Man to train patients’ upper arms and telemonitored by an occupational therapist.	Cost effectiveness analysis comparing (i) Conventional Occupational Therapy (COT); (ii) RAT used in clinic; (iii) RAT used at home. N = 12 (Home RAT) Mean age 59.4 ± 9.5 years (Home RAT) Data for comparison are taken from a previous RCT. Stroke in more than >28 days ago before treatment.	Home RAT over 30 days: 20–30 min per session/day to 60 min/day in week 1. Week 2, the training was up to 120 min/daily. COT: 18 × 90-min sessions for 6 weeks, 3x/week, a 90 min-duration match in-clinic therapy supervised by a trained OT. In clinic RAT: 18 × 90-min sessions for 6 weeks, 3x/week of in-clinic supervised RAT with 60 min of H-Man and 30 min COT session.	CEA using Fugl-Mayer Assessment (FMA) scores. ICERs reported.	Clinical outcome: the adjusted predicted mean (± SD) of FMA: Home-RAT = 45.8 (± 14.1) vs COT = 40.4 (± 11.6) vs in-clinic-RAT =45.3 (± 11.4). Lower mean healthcare cost was observed from home-RAT (Home-RAT: S$ 2416.9 vs COT: S$ 3191 vs in clinic RAT: S$ 3282.1).	Home-RAT was cost effective as compared to (i) COT and (ii) RAT in clinic.	A small sample size. Comparison groups were drawn from the previous RCT. It is not clear if the duration of the COT and clinic-based RAT are the same in practice to those used in the RCT.
Arbuckle et al. 2025. USA	To evaluate the delivery of high dose neurorehabilitation through both synchronous and asynchronous training using MindMotion Go to train not limited to upper limb impairment with the presence of a therapist for 30 min during synchronous session.	Cost analysis. N = 17 Mean age 54.8 ± 14.1 years Chronic stroke having a first-time stroke ≥3 months previously.	One intervention group to perform synchronous and asynchronous telerehabilitation. Training dose: 36 h or at minimum 120 min/week for duration 12–18 weeks. 1.Synchronous sessions with the presence of a therapist through visual observation for 30 min to have discussions on the training goals, feedback, adjustments, etc. 2. Asynchronous sessions is a self-gamified training by patients at home.	Cost analysis based on therapist time using hourly salary.	Cost per patient was USD 338 compared to USD 1903 for direct supervision.	A high dose asynchronous telerehabilitation addressed the problem with the shortage or limited amount of therapist time using a gamified technology driven method. It was also observed to be a resource cost-effective.	Small sample size. The cost analysis was limited to the cost of delivery - no other costs were included. The cost of comparator was based on the cumulative training dose had a therapist been present.
Cano-Mañas et al. 2020. Spain	To determine the effects of a structured protocol using commercial video games on balance, postural control, functionality, quality of life, and level of motivation in patients with a subacute stroke	RCT. N = 48 mean age 63.13 ± 10.38 Stroke in the subacute phase of illness, 15 days - six months after the vascular event	(i) 8 weeks of conventional rehabilitation (5 weekly sessions each 35 min) + video-game (20 min) using Xbox 360 game console and the Kinect device (3 weekly sessions). (ii) 8 weeks of conventional therapy (5 weekly sessions each 45 min) - 40 of PT, 40 of OT	EQ-5D-3L collected at baseline and 8 weeks after the intervention	Statistically significance differences were found between groups in the pain/discomfort domain (p < 0.01), and anxiety/depression domain (p < 0.01) and the VAS (p < 0.01)	A protocol of semi-immersive video-game based therapy, combined with conventional therapy, may be effective for improving balance, functionality, quality of life, and motivation in patients with subacute stroke	No cost or cost effectiveness analyses undertaken No tariff applied to EQ-5D domains Small sample size
Chen et al. 2022. China	To preliminarily prove the concept that combining mirror therapy and gamification concepts in VR can improve the functional performance of stroke patients.	Before and after study N = 48 Mean age not given categories age range 31–90 Stroke survivors minimum two months post stroke	Rehago, a gamified mirror therapy used during their session with therapist supervision. 30 minutes training per day, 5 days per week over a course of 6 weeks. No control	EQ-5D-5L collected at baseline to the post-treatment of Rehago at day 42.	VAS scores showed statistically significant (p < 0.01) increase between baseline and day 42 (69.65 ± 17.63; 76.38 ± 13.61) The mean 5L domain score decreased (12.52 ± 4.19 - 11.62 ± 3.93) (p < 0.05).	An improvement of the FIM score and the quality-of-life score in EQ5D-5L was observed, indicating it is beneficial to the patients using Rehago as a home-based rehabilitation tool.	No cost or cost effectiveness analyses undertaken No tariff applied to EQ-5D domains Small sample size No control group
Choi et al. 2016. China	To develop a mobile game-based upper extremity VR program for patients who have experienced stroke, and to evaluate the feasibility and effectiveness of the program.	RCT N = 24 Mean age intervention 61.0 ± 15.2 years; control 72.1 ± 9.9 years Diagnosis of an ischemic stroke. The results indicate ∼15 months stroke duration	(i) 30 min of conventional occupational therapy (OT) and 30 min of the mobile upper extremity rehabilitation program using a smartphone and a tablet PC (MoU-Rehab) (ii) 60 min of standard OT Both 10 sessions of therapy, 5 days per week, for 2 weeks.	EQ-5D collected at baseline, end of treatment and 1 month follow up. User satisfaction survey which asked how much you would be willing to pay for this program.	The mean 3L domain score showed no statistically significant differences between groups Respondents were willing to pay $22 +/−10 (between $10–40) for MoU-Rehab.	This mobile game-based VR rehabilitation program appears to be feasible and effective for promoting upper limb recovery after ischemic stroke.	No cost or cost effectiveness analyses undertaken. No tariff applied to EQ-5D domains Small sample size
Hesse et al. 2014. Germany	To evaluate the effectiveness and efficiency of robot-assisted arm group therapy (RAGT) versus individual arm therapy (IAT) to restore motor function in the moderately to severely affected patient after stroke.	RCT N = 46 Mean age intervention 71.4 (SD 15.5); control 69.7 (SD16.6) First-time supratentorial stroke (haemorrhagic or ischemic), lesion interval < eight weeks.	(i) 30 min of RAGT + 30 min of IAT for four weeks (ii) 2 × 30 min of IAT per workday for four weeks.	Cost analysis based on prices of the devices, employer's costs of staff and the mean number of patients treated in the arm studio or by a single therapist in one year.	The annual total costs of RAGT and salary accounted 37,000 euros or 4.15 euro per treatment. IAT costs 38,500 euros per annum or 10 euros per treatment, suggesting that the RAGT cost was lower by 5.85 euro per session.	RAGT in combination with IAT was equally effective as a double session of IAT regarding the restoration of upper limb motor functions in moderate to severely affected subacute patients with stroke. The treatment costs for RAGT were less.	Small sample size It is not clear if comparator is usual care The cost analysis was limited to the cost of delivery (including equipment) - no other costs were included.
Ho et al. 2019. Taiwan	To assess the impact of adjuvant VR technology on multidimensional therapy for patients with acute-stage stroke.	Cost analyses alongside prospective study with matched controls N = 200 Mean age VR + CT 67.97 (SD11.38); CT 67.68 (SD11.13) Diagnosis of acute ischemic stroke with the onset time < 7 days; minor to moderate stroke severity as defined by the National Institutes of Health Stroke Scale (NIHSS) score ≦ 16;	(i) Combined adjuvant VR-based rehabilitation program and conventional therapy (VR + CT). VR 20 min; CT 40 min daily for one week (ii) CT alone.	Cost analyses used medical cost data billed by the National Health Insurance on behalf of each patient across the hospitalisation period	VR + CT costs were statistically significantly lower TWD49,474 vs. 66,306, P < 0.005	The combination of VR-based rehabilitation and traditional therapy could be more effective for neurorehabilitation than CT alone in the early improvement of symptom severity, functional outcomes, and lower medical expenditure in acute stroke patients.	Voluntary selection of treatment may introduce bias between groups. Short intervention period (1 week) and lack of post-experiment follow up.
Islam, et al. 2019. Denmark, Norway Belgium	To illustrate the cost savings of VR training with different scenarios based on data from the VIRTUES trial.	Cost analyses based on data from multi-country RCT N = 120 No details of mean age of participants in the RCT Within 3 months post-stroke	4 weeks of 4-5 sessions of 45–60 min either: (i) VR training with YouGrabber system (ii) Individually tailored conventional upper extremity training (CT)	Cost analyses based on data from VIRTUES trial including cost of: therapist salary, device, kits for CT. Cost scenarios presented altering therapist time	N = 120 CT with 1:1 supervision $65,487 VIRTUES N = 60 CT; N = 60 VR with 1:1 supervision cost reduced to $15,088 Sliding scale presented with increase in proportion of patients using VR and VR assumed only to require 25% of the time used in CT. All scenarios show cost savings	VR incurs extra costs as compared with CT when the same amount of therapist contact is provided. However, these costs may be counterbalanced when time for therapist supervision can be reduced.	Authors cite speculative nature of reduction in therapist time with no evidence of cost effectiveness. The cost analysis was limited to the cost of delivery (including equipment) - no other costs were included.
Johnson et al. 2018. Australia	To investigate the STRoke Interactive Virtual thErapy (STRIVE) intervention on upper-extremity clinical outcomes in community-dwelling stroke survivors	RCT N = 58 Mean age 61.4 (SD 8.7) Time post stroke 13.4 (SD 8.9)	8 weeks of either (i) virtual therapy (VT) consisting of approximately 45 min of twice weekly VT training on the Jintronix Rehabilitation System (ii) usual care	EQ-5D-5L collected at baseline and end of treatment.	No changes were observed in the EQ-5D-5L index overtime for the VT (pre 0.53 +/− 0.14 to post 0.56 +/−0.18, P = 0.409, d = 0.00) or UC (pre 0.56 +/−0.12 to post 0.53 +/− 0.16, P = 0.422, d = 0.00).	No improvement or changes were seen in the health-related quality of life over five dimensions: mobility, self-care, usual activities, pain/discomfort and anxiety/depression	Whilst no improvement in QoL the VT exercise programme enhanced the UE function. Reduction in the spasticity on shoulder and elbow were observed but there was lack of improvement in hand and wrist functions. Small sample size
Rodríguez-Hernández et al. 2021. Spain	To evaluate the influence of conventional rehabilitation combined with virtual reality on improving quality of life related to post-stroke health.	RCT N = 43 Mean age 62.6 (SD 13.5) Sub-acute stroke. Intervention group time since diagnosis (ischemic/thrombotic) mean 91.3 days, control 85 days; time since diagnosis (hemorrhagic) intervention mean 8.7 days; control mean 10 days	Both groups 15 sessions over 3 weeks: (i) 100 min of physio and occupational therapy + 50 min of VR-based physio and occupational therapy (Hand Tutor, 3D tutor, Rehametrics software and Microsoft Kinect sensor) (ii) 75 min of conventional standard rehabilitation therapy and 75 min of occupational therapy	EQ-5D-5L collected at baseline, post-intervention and 3 month follow up	EQ-VAS score was reported: Intervention group 29.1 ± 12.8 at baseline, 86.5 ± 7.1 post-intervention, and 78.3 ± 10.3 at the three-month follow-up. The control group obtained scores of 25.5 ± 5.1, 57.0 ± 4.7, and 58.5 ± 5.9, respectively. Statistically significant differences between post intervention and follow up. Proportion of participants in each level of each domain reported	A combined conventional and virtual semi-immersive based therapy is more effective than the conventional therapy to improve the self-perceived health-related quality of life (HRQoL) and reduce severity of the dimensions of the EQ-5D-5L. Three months after the treatment, the effect of the combined intervention decreased in the pain and anxiety dimensions.	The EQ-5D tariff not applied for single score. The therapist assigned to the conventional rehabilitation therapy was blinded but the intervention was not blinded to the participants and combined treatment therapist
McCabe, J. et al. 2015. USA	To compare response to upper-limb treatment using robotics plus motor learning (ML) versus functional electrical stimulation (FES) plus ML versus ML alone, according to a measure of complex functional everyday tasks for chronic, severely impaired stroke survivors.	Cost analyses alongside a randomised trial N = 35 No details of participants age given >1 year post-stroke	All groups received 5 h/day, 5 days per week, 12 weeks (60 sessions) (i)FES plus ML (3.5 h/d of ML and 1.5 h/d of FES wrist/hand coordination training) (ii)ML (5 h/d partial- and whole-task practice of complex functional tasks) (iii)robotics plus ML (3.5 h/d of ML and 1.5 h/d of shoulder/elbow robotics)	Costs analyses included therapist time, robot costs (including maintenance), FES cost to calculate per patient cost per protocol	ML alone ($4570), FES plus ML ($4604), and robotics plus ML ($5686).	Severely impaired stroke survivors with persistent (>1y) upper-extremity dysfunction can make clinically and statistically significant gains in coordination and functional task performance in response to robotics plus ML, FES plus ML, and ML alone in an intensive and long-duration intervention	The cost analysis was limited to the cost of delivery (including equipment) - no other costs were included. Small sample size and no details of participants demographics
Masiero et al. 2014. Italy	To evaluate the costs related to delivering the Neuro-RehabilitationRobot (NeReBot) for the treatment of poststroke upper limb impairment, with reference to the standard costs of stroke rehabilitation in the Italian National Health Care System	Cost analysis using data from 2 RCTs No details given re age or sample size. N = 35 (The robotic treatment in addition to the conventional therapy) N = 21 (Dose-matched robotic therapy) Hemiparetic participants in the acute and subacute phases of their stroke, enrolled within 15 days after stroke	The 2 RCTs tested two different robotic protocols in comparison to standard rehabilitation treatment, one using the robot in addition to the traditional treatment, one in partial substitution to the standard rehabilitation programme, with a dosematched approach. Both protocols lasted 5 weeks and included two daily sessions of robotic treatment for five days a week week	Cost analyses included costs of robot and therapist Costs calculated for 4 different levels of supervision (from 1:1 to 1:4, i.e., 30, 15, 10, and 7.5 min per session).	Hourly treatment cost of robotic therapy ranged between €25 and €11 1) Total additional protocol costs with 4.17 h/week (25 min x 2/d x 5d/week) = €328.04/patient. Estimated to reduce 1.2 days patient's hospitalisation or discharging patient 2 days earlier. One day hospitalisation costs €273.64. Standard UL rehab: 40 h for 8 weeks. 2) Total Mixed Protocol Cost: Phase I: Additional robotic therapy for 2 weeks for 20 sessions = €170.21/patient Phase II: robotic treatment substituted the conventional therapy, equivalent to 24.49 h. Hourly saving €7/patient (therapist cost: €18-€11 hly robotic training cost for ratio 1:3 to 1:4). Substitution weeks accounted for 5.78 weeks for 58.78 sessions.	In the proposed mixed protocol, without any additional cost with respect to conventional rehabilitation, patients would receive, in the subacute phase, more than 20% additional treatment time (8 h) with respect to the one normally delivered to the proximal upper limb (40 h within 8 weeks). The intensification of treatment in the first weeks is expected to bring greater gains on both functional and motor scales, on the basis of the results of the first clinical study	Authors note estimates assume access to more than one robot and a full cost effectiveness analysis is needed Other than the estimate of reduce hospital stay, the cost in one scenario, the analysis was limited to the cost of delivery (including robot) Sample size in the 2 RCTs was small
Vanoglio et al. 2017. Italy	To evaluate the feasibility and efficacy of robot-assisted hand rehabilitation in improving arm function abilities in sub-acute hemiplegic patients.	Cost analyses alongside RCT N = 27 Mean age intervention 72 (SD11); control 73 (SD14) Patients affected by stroke from cerebral ischemia or hemorrhage that had occurred ⩽ 30 days before	Rehabilitation began day after admission and continued for around 6 weeks - 30 sessions 40 min/day, 5 days per week (i) Intensive hand training with Gloreha, a hand rehabilitation glove that provides computer-controlled, repetitive, passive mobilisation of the fingers, with multisensory feedback. (ii) Conventional hand rehabilitation.	Costs analyses included therapist time, robot costs (including depreciation),	Cost analysis: Intervention cost of physion€147.60 per patient. Cost of device €89.60 per patient Conventional therapy: € 480 per patient. Physiotherapist time commitment of 3 days vs 27 days: 24 (8.5) vs 11 (1.1).	The robotic hand rehabilitation glove showed efficacy in improving the motoric arm function, paretic upper limb (MI), their coordination mono-manual dexterity (NHPT), strength (G&P Test). The robot-assisted hand therapy was around half of the cost of the conventional rehabilitation. The results suggest that is more cost-effective and superior to the conventional therapy.	The cost analysis was limited to the cost of delivery (including equipment) - no other costs were included. Small sample size
Valles et al. 2016. Mexico	To develop and deliver an effective and cost and labour efficient method of post-stroke rehabilitation that encouraged continued rehabilitation and was more or as effective as traditional physical and occupational therapy approaches	Cost analysis alongside a pilot RCT N = 20 Mean age robotic 44.1 ± 12.55; control 64.1 ± 8.38 Clinically diagnosed with hemiplegia from a stroke occurred more than 6 months prior the study	24 two-hour therapy sessions over 6 to 8 weeks (i) Robot Gym therapy (4 stations per session). Of 6 stations, 3 were for upper extremities rehabilitation, 2 for lower limb, and 1 for cognitive training. (ii) 2-h standard traditional therapy session (including personalised physical and occupational sessions) with one-to-one therapist to patient ratio.	Total treatment operating expenses or the sum of the maintenance equipment cost and employing therapist at yearly basis by total number of patients handled by a therapist per annum.	The therapy cost per session was $230.52 MXN or $USD 19.21. While for the Robotic Gym cost was $83,90 MXN or $USD6.99 in the first 2 years then reduced subsequently to $51.48 MXN or $USD 4.29 per session.	Robot Gym can be as effective as traditional therapy for stroke patients, presenting a more cost- and labour-efficient option for countries with scarce clinical resources and funding	Comparator was not usual care and the cost analysis was limited to the cost of delivery (including equipment) - no other costs were included. Small sample size

**Abbreviations:** RCT = randomised controlled trial; CEA = cost-effectiveness analysis; ICER = incremental cost-effectiveness ratio; RAT = robot-assisted therapy; COT = conventional occupational therapy; FMA = Fugl-Meyer Assessment; VAS = visual analogue scale; HRQoL = health-related quality of life; ML = motor learning; FES = functional electrical stimulation; IAT = individual arm therapy; RAGT = robot-assisted group therapy; NIHSS = National Institutes of Health Stroke Scale; UL = upper limb; OT = occupational therapy; PT = physiotherapy; CT = conventional therapy; SD = standard deviation; FIM = Functional Independence Measure; MI = Motricity Index; NHPT = Nine-Hole Peg Test; G&P Test = Grip and Pinch Test; VR = virtual reality.

Across the included studies, clinical effectiveness was most commonly assessed using upper limb motor function measures, including the Fugl-Meyer assessment (FMA), Motricity index (MI), Nine-Hole Peg Test (NHPT), and other task-based functional scales. Some studies also reported broader clinical measures, such as the National Institutes of Health Stroke Scale (NIHSS). In addition, six studies collected preference-based measures, primarily the EQ-5D, to assess health-related quality of life. The EQ-5D generates utility values that can be used to estimate QALYs for cost-effectiveness analysis.

### Interventions

The interventions were either VR-based or robotic-aided rehabilitation.

Approximately eight studies (53.33%) engaged with VR-based training, including immersive Nintendo Wii video game,^15^ interactive self-select VR training,^9^ wearable gloves such as the BiManu trainer,^7^ Hand Tutor glove and 3D Tutors motor training,^14^ and other VR-based game therapies, such as Rehago,^10^ mobile-based game applications,^16^ and interactive VR-based training.^8,17^ In one study, the authors examined the implementation of combined VR-based devices and conventional rehabilitation and their effects on upper and lower limb strength and motor functions.^18^

Seven studies (46.67%) report robotic aided exercises.^11–13^^,18–21^ In two of these studies, interventions were delivered in an arm studio consisting of six stations or devices under a supervising therapist where each patient who underwent treatment moved from one station to another in a single training session. In one of these studies, patients completed the robotic-assisted therapy focusing on improving the UL paresis^20^; whilst in the other study patients were treated to train their upper and lower body functions.^13^

The duration of intervention across the studies ranged from 20 min to 2 h per session. Whilst the total duration of the programme for all studies lasted from 1 to 18 weeks, 80% of them were between 4 and 18 weeks.^8,11,12,18^

Individuals taking part in the studies underwent a variety of frequencies and types of treatment protocols. Participants received combined training of VR-based or robotic-aided therapy and standard rehabilitation in 40% of the studies.^8,14,16,17,20,21^ The remaining 60% of studies either delivered the intervention separately between the treatment and control groups^7,9,11,13,15,18,19^ or had a single-arm intervention without a control group.^10,12^ One study introduced two protocols for two different sets of treatment and control groups.^21^ In the first protocol, the robotic device (NeReBot) was given as an adjunct treatment on top of the standard rehabilitation while in the second group, NeReBot was given as a partial substitution to the usual care. A single arm study evaluated a high-dose neurorehabilitation programme that used self-training technologies at home under remote therapist supervision.^12^

The level of therapist involvement also differed across studies. Some interventions were primarily home-based and self-directed, in certain cases supported by remote monitoring or limited synchronous contact, whereas others were delivered under direct therapist supervision in clinic-based or group settings. These differences in supervision intensity are relevant when interpreting the reported costs, particularly where personnel time formed a substantial component of the cost estimates.

### Cost effectiveness analysis

Of two studies employing cost-effectiveness analyses, only one estimated that the intervention was cost-effective. Adie and colleagues compared home-based use of a Nintendo Wii to improve arm function after stroke with conventional arm exercises.^15^ No difference was found in QALYs, and costs were higher in the Wii group, meaning that the intervention was not cost-effective. Whilst the perspective of the analysis was not explicit, overall, the analysis is clear and well reported. Aguirre–Ollinger and colleagues assessed the feasibility of a 2-D planar arm, a home-based robot-assisted telerehabilitation (RAT), among post-stroke survivors in Singapore.^11^ A cost-effectiveness analysis was conducted from a societal perspective (patient and hospital costs). The analysis uses the data from the study together with data from a previous randomised controlled trial (RCT) to compare the RAT at home, RAT used in clinic and conventional occupational therapy (COT). Cost savings and a positive incremental effect were reported, thus, RAT at home dominated (i.e., lower costs and greater health benefits), indicating cost-effectiveness.

### Cost Analysis

Of the eight cost studies, five showed lower costs in the intervention arm.^7,8,11,17,18^ However, six studies reported only the cost of delivery (defined as the cost of the device or other equipment). The exception, Ho et al.,^8^ included medical cost data billed by the National Health Insurance on behalf of each patient across the hospitalisation period. Whilst the study is a cost analysis rather than a cost-effectiveness analysis, the authors concluded that the combined virtual and conventional therapy was more cost effective than the single conventional rehabilitation. This was largely due to a lower cost, while exhibiting better clinical benefits of improved functional recovery outcomes and reduced stroke severity (referring to previous work on the clinical outcome). The combined virtual and standard rehabilitation cost was TW$49,473.66 or TW$16,832 lower than the traditional care (p = 0.042). However, the study is limited by a short intervention period (1 week) and lack of post-intervention follow up.

Valles et al.^13^ suggest the use of Robot Gym is cost-effective. They undertook a cost analysis alongside a pilot RCT comparing Robot Gym therapy with conventional care. They estimated an intervention per patient treatment cost of US$6.99 per session in the first 2 years, which reduced to US$4.29 per session in the subsequent years. In comparison, the standard care per patient per treatment cost was US$19.21. However, the pilot study was not powered to show clinical effectiveness and should be viewed as exploratory. The cost analysis was also limited to the purchase of the device and treatment delivery.

Of the other studies that estimated lower costs, Hesse et al.^20^ undertook a cost analysis alongside an RCT of robotic arm assisted group therapy (compared with individual arm therapy) and found the intervention cost lower by €5.85 per session. The sample size is modest in the RCT (n = 46), and cost estimates were limited to cost of delivery (including equipment). In their evaluation of the efficacy of robotic assisted hand rehabilitation compared with conventional hand rehabilitation, Vanoglio et al.^19^ estimate the cost of the intervention to be around half that of conventional therapy (€237.60 vs €480.00). Again, this was based on modest participant numbers (n = 27) and the estimated costs included only delivery (therapist time, robot, and depreciation). Islam and Brunner^7^ used different scenarios to illustrate the cost savings of VR training varying the proportion of patients using VR, reporting a reduction in therapist time by 75%. It was assumed that although VR incurs extra costs when compared with conventional therapy, these costs may be counterbalanced when time for therapist supervision can be reduced. In their study the sample size is larger (n = 120), but the scenarios are somewhat speculative, and again the cost estimates are limited to delivery (including equipment).

McCabe et al.^18^ explore the comparison of motor learning methods (ML) alone, ML plus functional electrical stimulation (FES), and robotics plus ML. None are considered usual care. The costs of the treatment protocols were $4570, $4604, and $5686 respectively. The authors concluded that if a cost differential of approximately $1000 per patient is considered important, then the FES plus ML protocol and/or the ML alone protocol would be preferable. The estimates are based on a small sample (n = 35) and there is no comparison with usual care which makes it difficult to place the estimates in context of the cost of current clinical practice.

The final cost study, Masiero et al.,^21^ seeks to evaluate costs related to delivering the Neuro-Rehabilitation Robot (NeReBot) with reference to the standard costs of stroke rehabilitation in Italy. In this study, the authors modelled different scenarios with varying levels of supervision. They proposed a mixed protocol in which patients received more treatment time with the expectation that the intensification of treatment in the first weeks would bring greater patient gains. The expectation of gains is based on the results of one RCT with a relatively small sample size.

### Preference-Based Measures

Six studies used preference-based measures. A preference-based measure is a tool to assess health-related quality of life and to generate utility values that can be used to estimate QALYs in cost-effectiveness analysis. These measures allow comparison of the value of different interventions, services, or programmes and can inform resource allocation decisions where resources are limited.

Adie et al.^15^ compared the home-based use of the Nintendo Wii to conventional arm exercises. The VAS in their cost-effectiveness analysis shows improvements in both intervention and control over time (baseline, 6 weeks, 6 months), but there is no significant difference between the groups. Whilst it does not explicitly report the EQ-5D-3L value based on the tariff, the paper reports the QALYs derived from these figures for the cost-effectiveness analysis. There was no difference between intervention and control in QALYs reported.

Cano-Mañas and colleagues^17^ employed an RCT approach to compare structured video games and conventional therapy. Data collection included the EQ-5D-3L. Unlike the other studies, no tariff is applied to derive a single figure. The VAS is reported together with the individual domains. The three levels in each domain are ranked and a mean score presented. Within the control group, statistically significant improvements over time were found in the anxiety/depression domain (p = 0.03); in the intervention group there was significant improvement in the pain/discomfort domain (p < 0.01). Between the two groups statistically significant differences were found in pain/discomfort and anxiety/depression domains and the VAS (all p < 0.01). Based on these results together with the other measures collected, the authors concluded video-based game therapy combined with conventional therapy might be effective.

Chen et al.^10^ in their pilot RCT collected EQ-5D-5L data at baseline and 42 days. The before and after study of gamified mirror therapy reported an increase in the VAS over the time-period. Like the previous work,^15^ they rank the domains (in this case 1–5 where 1 is more independent and 5 is more dependent) summing them to produce a mean figure which decreased over the time period and was statistically significant.

Choi et al.^16^ collected EQ-5D-3L data within their RCT to evaluate mobile game-based VR. They refer to the EQ-5D index, but the figures reported do not suggest that the relevant preference-based tariff has been used – rather a ranking has been employed that shows an improvement after treatment in both groups. No statistically significant difference was found between groups.

A recent study from Rodriguez-Hernandez and colleagues^14^ collected EQ-5D-5L data at baseline post intervention and three months within their RCT of combined conventional and semi-immersive VR therapy. The EQ-VAS score was reported showing statistically significant differences between groups. In addition, the proportion of participants in each level of each domain was reported. The authors conclude VR was more effective than conventional therapy to improve health-related quality of life (HRQoL) and reduce the severity dimension in the pain/anxiety domain.

Based on the assessment using the completed critical appraisal form, studies varied in terms of methodological rigour. Whilst the overall quality of the papers was mixed, most studies reported clearly defined research questions and objectives, provided a comprehensive description of the comparators, and outlined their methods clearly. However, limitations were observed in the identification of important costs. Some studies conducted formal cost-effectiveness analyses with incremental comparisons, whereas many were limited to cost descriptions or reported preference-based outcomes without economic evaluation. Sensitivity analyses were infrequently undertaken.

## Discussion

Of the 15 included studies, two reported cost-effectiveness analyses,^11,15^ eight reported cost analyses,^7,8,12,13,18–21^ and six reported use of a preference-based measure (in all cases the EQ-5D).^9,10,14–17^

The cost-effectiveness analyses reported conflicting results, with one finding the intervention cost-effective and one not. Aguirre–Ollinger and colleagues^11^ put forward two possible explanations for the difference between their results and those of Adie et al.^15^ The first is the choice of comparator and the second is the limited capabilities of using a commercial gaming console. However, their study data for the intervention were limited by a small sample size (n = 12). Whilst the lower costs were driven by reduced staff time, the RAT in clinic was over 6 weeks compared to use at home of 30 days; it is not clear if in practice it would be anticipated that the duration of use would be the same for each.

None of the cost analysis-related studies attempted to combine the cost and effectiveness results. Indeed, the majority of the RCTs were exploratory in nature, with ≤ 50 participants.^12,18–21^ Only two studies had larger samples (n = 120^7^ and 200^8^). Ho et al.^8^ used retrospective cross-matching, which is prone to some validity issues owing to the lack of a control group.^22^ Islam and Brunner^7^ also modelled reductions in therapist time.

Overall, whilst the evidence is mixed, the papers reviewed show that these types of intervention have promise in respect of cost savings for rehabilitation training for upper limb function after stroke. The included articles suggest that adequate uptake by patients receiving technology-based treatment is required to compensate for the capital costs incurred in investing in telerehabilitation technologies such as VR or robotic-based therapy.^7^ As post-stroke telerehabilitation care mostly offers stroke survivors home-based or self-operated devices, contacts with in-person therapists as seen in the delivery of clinic-based rehabilitation have the potential to be reduced. Indeed, two studies modelled reductions in therapist time in their cost estimates.^7,21^ A smaller patient-to-therapist ratio can lead to a decrease in therapist time, and this wage reduction may be sufficient to offset the investment cost of devices. Reductions in therapist time were more commonly modelled in home-based or partially supervised interventions, where technology reduced the need for continuous one-to-one contact.^7,12,21^ In clinic-based robotic programmes, cost differences were more often related to group delivery or altered staff-to-patient ratios rather than complete replacement of therapist input.^13,20^ This suggests that cost savings are closely linked to the mode of delivery.

However, few of the papers considered costs to the health and social care system outside the delivery protocol, and longer-term effectiveness or cost-effectiveness was rarely evaluated. Most studies had short follow-up periods and therefore did not capture potential longer-term costs or savings associated with sustained use of technology-based rehabilitation. As a result, conclusions regarding long-term cost-effectiveness remain uncertain.

These findings are consistent with previous reviews suggesting that telerehabilitation can achieve comparable clinical outcomes to in-person therapy, although economic evidence remains limited. While earlier reviews have focused primarily on effectiveness, our review highlights the limited number of full cost-effectiveness analyses and the inconsistent reporting of utility values. Taken together, these gaps highlight the need for robust and standardised economic evaluation in this field.

Six studies in the review reported the use of a preference-based measure, in all cases the EQ-5D.^9,10,14–17^ However, few studies converted EQ-5D responses into utility index values using published tariffs. Most reported only VAS scores or domain-level results rather than utility index values, and reporting across studies was inconsistent. This limits interpretation of the findings in terms of cost-effectiveness, as utility values are required to estimate QALYs. The results were mixed. One study did not observe any change in EQ-5D-5L scores among treated patients over time; two other studies found no difference between treated and control groups despite improvement over time in both groups.^9,15,16^ Three studies noted differences between groups indicating effectiveness.^10,17^ With the exception of Adie et al.,^15^ all studies were relatively modest in size (≤60 participants).

The review has been carried out in line with PRISMA guidelines. For completeness, interventions designed for lower and upper limb were included. We were unable to extrapolate costs related only to upper limb rehabilitation. However, their inclusion does not change the interpretation of the overall results.

There are several limitations that should be considered when interpreting the findings of this review. First, most included studies had small sample sizes and were exploratory in nature. Second, follow-up periods were generally short, limiting conclusions regarding long-term cost-effectiveness. In addition, reporting of utility values and economic outcomes was inconsistent across studies.

To conclude, current evidence on the cost of delivery of telerehabilitation training for upper limb function in people after stroke is focused on therapist time and the cost of the equipment and/or device. Reductions in therapist time resulting from the use of telerehabilitation training have the potential to offset or exceed the cost of the technology or equipment. Costing equipment or new technologies should consider the inclusion of not only the cost of the device but also additional costs including ongoing maintenance, depreciation, internet access and laptops.

None of the literature addressed the cost of implementation, nor was there clear evidence of cost-effectiveness. In line with UK guidance, cost-effectiveness analysis should use a preference-based measure such as the EQ-5D. Our review found that whilst almost half the studies included the EQ-5D, few reported utility values, instead reporting VAS values.

## Clinical messages

- Use of VR-based or robotic-aided rehabilitation may support delivery of upper limb rehabilitation following stroke, with potential cost implications influenced by therapist time and mode of delivery.
- Reductions in therapist time may help offset the cost of equipment, but evidence of full cost-effectiveness remains limited.
- More evidence is needed on the cost-effectiveness of VR-based or robotic-aided rehabilitation for this population, including potential resource savings over the longer term.

## Supplemental Material

sj-docx-1-cre-10.1177_02692155261441561 - Supplemental material for Costs and cost effectiveness of the use of telerehabilitation training for upper limb function in people after stroke: A systematic review Supplemental material, sj-docx-1-cre-10.1177_02692155261441561 for Costs and cost effectiveness of the use of telerehabilitation training for upper limb function in people after stroke: A systematic review by Daim Syukriyah, Jemma Perks, Phil McBride, Philip Clatworthy, Helen Dawes, Maedeh Mansoubi, Tristan Snowsill, Gordon Taylor and Claire Hulme in Clinical Rehabilitation

## Notes

a. Total original patients participated in 15 studies initially were 1,034 people but only 963 completed the studies.
