# Supplementary material for: Costs and cost effectiveness of the use of telerehabilitation training for upper limb function in people after stroke: A systematic review
Source: Clin Rehabil. 2026 May 2;40(11):1423–37. doi: 10.1177/02692155261441561 (PMC13736509; doi:10.1177/02692155261441561)
Supplement: Supplementary file 1 [file sj-docx-1-cre-10.1177_02692155261441561.docx]

**Supplementary Material**

**Supplementary Appendix 1. MEDLINE search strategy**

1. (stroke or cerebrovascular accident$ or cerebrovascular insult$ or cerebr$ infarct$).ti,ab,kw.

2. exp Stroke/

3. (rehab$ or neurorehab$ or physiotherap$).ti,ab,kw.

4. (1 or 2) and 3

5. stroke rehabilitation/

6. 4 or 5

7. (hand or forearm or upper limb$).ti,ab,kw.

8. exp Upper Extremity/

9. 6 and 8

10. (virtual realit$ or augmented realit$ or mixed realit$ or virtual environment$ or immersive environment$).ti,ab,kw.

11. VR.ti,ab,kw.

12. (video gam$ or exergam$ or serious gam$ or gamification or kinect or nintendo or wii or playstation or oculus or meta quest).ti,ab,kw.

13. (skype or zoom or microsoft teams or google meet or google hangout$ or videoconferenc$ or video chat or video call$).ti,ab,kw.

14. (telephon$ or phone or teleconferenc$ or tele conferenc$ or telerehab$ or tele-rehab$).ti,ab,kw.

15. exp virtual reality/

16. Computer-Assisted Instruction/

17. exp Telemedicine/

18. Videoconferencing/

19. or/10 –18

20. 9 and 19

**Supplementary Appendix 2. Summary of critical appraisal (CASP)**

| **Study** | **Clear Research Question** | **Appropriate design** | **Costs adequately identified** | **Outcomes appropriately measured** | **Incremental analysis conducted** | **Sensitivity analysis conducted** | **Key limitation** |
| --- | --- | --- | --- | --- | --- | --- | --- |
| Adie et al. 2017. UK | Yes | Yes (RCT & CEA) | Yes (NHS & intervention costs identified; short time horizon) | Yes (QALYs via EQ-5D-3L tariff) | Yes (ICER calculated) | Yes (bootstrapping & parameter uncertainty) | Short follow-up (6 months); limited time horizon |
| Aguirre‑Ollinger et al. 2024. Singapore | Yes | Yes (cost-effectiveness analysis using study and RCT data) | Partial (patient and hospital costs included; limited time horizon and costing scope) | Yes (FMA scores used to estimate incremental effects) | Yes (incremental cost comparison with dominance reported) | No (no formal deterministic or probabilistic sensitivity analysis) | Very small sample; comparator data from previous RCT; limited costing scope |
| Arbuckle et al. 2025. USA | Yes | Partial (retrospective, single-arm cost analysis) | Partial (therapist time cost only; limited costing perspective) | Yes (clinical outcomes properly measured) | No | No | Small sample, delivery costs only, comparator hypothetical |
| Cano-Mañas et al. 2020. Spain | Yes | Yes (RCT) | Not applicable (no cost analysis) | Yes (EQ-5D-3L collected, domains reported) | No | No | No economic evaluation, no tariff applied |
| Chen et al. 2022. China | Yes | Partial (before-after, no control) | Not applicable | Yes (EQ-5D-5L collected) | No | No | No comparator, no economic evaluation |
| Choi et al. 2016. China | Yes | Yes (RCT) | Not applicable | Yes (EQ-5D collected) | No | No | No economic evaluation, small sample |
| Hesse et al. 2014. Germany | Yes | Yes (RCT + cost analysis) | Partial (delivery costs only) | Yes (clinical outcomes measured) | No | No | Limited costing perspective |
| Ho et al. 2019. Taiwan | Yes | Partial (matched observational study and cost analysis) | Partial (hospital costs only) | Yes (clinical and cost outcomes) | No | No | Non-randomised, short duration |
| Islam et al. 2019. Denmark, Norway Belgium | Yes | Yes (scenario-based cost analysis using RCT data) | Yes (therapist time and equipment modelled) | Yes (clinical data from RCT) | Yes (cost comparison across scenarios) | Yes (scenario analysis only) | Assumptions regarding therapist time reduction |
| Johnson et al. 2018. Australia | Yes | Yes (RCT) | Not applicable | Yes (EQ-5D-5L index reported) | No | No | No economic evaluation |
| Rodríguez-Hernández et al. 2021. Spain | Yes | Yes (RCT) | Not applicable | Yes (EQ-5D-5L and VAS reported) | No | No | No tariff applied, no economic evaluation |
| McCabe, J. et al. 2015. USA | Yes | Yes (RCT and cost analysis) | Partial (protocol costs only) | Yes (clinical outcomes measured) | No | No | No comparison to usual care |
| Masiero et al. 2014. Italy | Yes | Yes (modelling and RCT data) | Partial (robot and therapist costs) | Yes (clinical referenced) | Yes (comparative cost scenarios) | Yes (scenario analysis only) | Model assumptions, limited cost scope |
| Vanoglio et al. 2017. Italy | Yes | Yes (RCT + cost analysis) | Partial (delivery costs only) | Yes (clinical outcomes measured) | No | No | Small sample, limited cost scope |
| Valles et al. 2016. Mexico | Yes | Yes (pilot RCT and cost analysis) | Partial (delivery costs only) | Yes (clinical outcomes measured) | No | No | Pilot study, comparator not usual care |

**Abbreviations:** CEA = cost-effectiveness analysis; ICER = incremental cost-effectiveness ratio; RCT = randomised controlled trial; FMA = Fugl-Meyer Assessment; EQ-5D = EuroQol 5-Dimension instrument; QALY = quality-adjusted life year.
